# Bariatric embolization with N-butyl cyanoacrylate glue: a revival

**DOI:** 10.1186/s42155-025-00602-6

**Published:** 2025-09-29

**Authors:** Romaric Loffroy

**Affiliations:** Department of Vascular and Interventional Radiology, Image-Guided Therapy Center, François-Mitterrand University Hospital, Dijon, France

## To the Editor

We read with great interest the article by Choi et al. recently published in *CVIR Endovascular* and comparing the efficacy and safety of N-butyl cyanoacrylate (NBCA) and microspheres in suppressing weight gain and ghrelin expression after bariatric arterial embolization (BAE) in a swine model [1].

We have several comments. First of all, we would like to congratulate the authors for their work which represents the first preclinical comparative study to date reporting results on the potential added value of NBCA in such a setting. Particulate bariatric arterial embolization (BAE) has been used over the last decade as an encouraging minimally invasive intervention for weight reduction in preclinical animal models as well as in clinical studies in patients with morbid obesity, with mixed results [2–5]. In the present study, the authors demonstrated that the NBCA group achieved significantly greater reductions in both weight gain and ghrelin expression in the stomach than those in the microsphere group in a swine model of BAE [1]. Another finding of utmost importance of this study was the lower rate (40% vs 100%) of superficial mucosal ulceration in the gastric fundus at the 1-week follow-up endoscopy, and the lower rate (0 out of 5 pigs, 0% vs 1 out of 5 pigs, 20%) of recanalization of the right and left gastric arteries at the 16-week follow-up angiography in the NBCA group compared to the microsphere group [1].

The work presented here is a great demonstration of how the general advantages of glue over particles can be applied to this particular indication. First, NBCA allows for fast embolization procedures, with lower radiation dose and cost [6, 7]. Second, the mixture of NBCA/Lipiodol® is highly radiopaque, making both the embolization process and end-point safer and more accurate by real-time assessment of injection and collaterals [8]. In addition, we can assume that Lipiodol uptake and distribution into the distal vascular bed could be used as a surrogate marker of outcomes on immediate post-embolization computed tomography scan of the abdomen. Third, its viscosity makes it highly penetrable but prevents passage through capillaries, thereby minimizing the risk of ischemic complications as shown previously for both upper and lower gastrointestinal bleedings, contrary to what most people think [6, 9, 10]. On this subject, we would be curious to know why the authors did not use a higher glue/Lipiodol ratio than 1:3? Indeed, although the brand name of NBCA used by the authors was not mentioned, we can suppose that they used Histoacryl®, Glubran®2 being not available in South Korea. Glubran®2, an NBCA with a comonomer (metacryloxysulfolane, MS) added to the glue, has a delayed polymerization time compared to Histoacryl®. It means that the standard ratio of 1:3 used with Glubran®2 probably corresponds to a higher ratio with Histoacryl®. That is exactly why we recommend the use of a higher ratio with pure NBCA. Fourth, the risk of recanalization is exceptional and the occlusive effect does not depend on coagulation parameters, leading to a more durable and efficient occlusion than particles [6, 7, 9]. In a word, we are in line with the authors on what glue can do best in such a setting. The only weakness of NBCA for bariatric embolization is possible pain during injection, supporting the use of MS-NBCA instead of NBCA given the lower exothermic reaction (45 °C vs 90 °C) and inflammatory response of the former.

In conclusion, these new data about the superiority of NBCA over particles for BAE in a preclinical model give hope that the technical advantages of glue can translate into clinical benefits, warrant the need for further clinical studies, and will maybe allow to revive the technique of BAE in obesity treatment in humans.

## Data Availability

Not applicable.
